# A Mouse Model of Chronic Pancreatitis Induced by an Alcohol and High Fat Diet

**DOI:** 10.2174/1876386301710010081

**Published:** 2017-09-15

**Authors:** T. Clinkinbeard, R.H. Kline, L.P. Zhang, S.L. McIlwrath, J.F. Watkins, K.N. Westlund

**Affiliations:** 1 Center for Gerontology, School of Public Health, University of Kentucky, 725 Rose St., Lexington, KY 40536, USA; 2 Department of Physiology, School of Medicine, University of Kentucky, 800 Rose St., Lexington, KY 40536-0298, USA

**Keywords:** Loperamide, Mu opioid, Hypersensitivity, Pain, Nociception, Pancreas, Fibrosis, Behavioral test

## Abstract

**Background/Aims:**

Study of acute pancreatitis in chemically-induced rodent models has provided useful data; models of alcoholic chronic pancreatitis have not been available in mice. The aim of the present study was to characterize a mouse model of chronic pancreatitis induced solely with an alcohol and high fat (AHF) diet.

**Methods:**

Mice were fed a liquid high fat diet containing 6% alcohol as well as a high fat supplement (57% total dietary fat) over a period of five months or as control, normal chow *ad libitum*. Pain related measures utilized as an index of pain included mechanical sensitivity of the hind paws determined using von Frey filaments and a smooth/rough textured plate. A modified hotplate test contributed information about higher order behavioral responses to visceral hypersensitivity. Mice underwent mechanical and thermal testing both with and without pharmacological treatment with a peripherally restricted μ-opioid receptor agonist, loperamide.

**Results:**

Mice on the AHF diet exhibited mechanical and heat hypersensitivity as well as fibrotic histology indicative of chronic pancreatitis. Low dose, peripherally restricted opiate loperamide attenuated both mechanical and heat hypersensitivity.

**Conclusion:**

Mice fed an alcohol and high fat diet develop histology consistent with chronic pancreatitis as well as opioid sensitive mechanical and heat hypersensitivity.

## 1. INTRODUCTION

In 2004, pancreatitis in the US cost an estimated $373.3 million in direct and indirect costs [1]. Eight in 100,000 people are diagnosed with chronic pancreatitis in the US yearly [2] and up to 50% of these individuals can live 20 years after diagnosis [3]. Fifty out of 100,000 people are living with chronic pancreatitis [4, 5]. Many of these patients endure severe intractable pain.

The development of chronic pancreatitis in humans can result from multiple possible contributing risk factors, one of which is the sensitizing effect of alcohol on the pancreatic cells [6]. Alcohol-activated local pancreatic reactive immune cells, the stellate cells, and their interaction with other pancreatic and invading immune cell types contribute to the overproduction of extracellular matrix proteins and pathogenesis of chronic pancreatitis that interferes with pancreatic functions [7 – 10].This overproduction occurs as pancreatic stellate cells attempt to repair damaged pancreatic cells [11]. It has been suggested that high levels of alcohol also decrease the solubility of proteins in the ductal space, leading to plugging of the ducts and the subsequent autodigestion of the pancreas [3].

The National Commission on Digestive Diseases (NCDD), under the umbrella of the National Institute of Diabetes and Digestion and Kidney Diseases (NIDDK), values the production of better animal models to further the study of chronic pancreatitis [12, 13]. Mouse models in which chronic pancreatitis is induced only with a combination of ad libitum high fat liquid food with added alcohol and lard supplementation do not currently exist. Current rodent models of pancreatitis induced by chemical irritants such as cerulein or dibutyltin dichloride (DBTC) have proven fruitful for studies of acute pancreatitis [14]. Acute models, however, do not provide the range of chronic pancreatitis symptoms seen in the clinic caused by predisposing risk factors. Likewise, a chronic model is more suitable for testing therapeutics for long-term, debilitating chronic pancreatitis pain.

In the present study, an alcohol and high fat diet (AHF) induced model of chronic pancreatitis is described based on a modified Lieber-DeCarli [15] diet containing 6% ethanol, added corn oil, and supplemental lard. This diet has been used with great success in rats to induce chronic pancreatitis within 4–5 weeks, confirmed using histology of the pancreas and pain-related behavioral studies [16 – 19]. The purpose of the present study was (1) to produce a diet-induced chronic pancreatitis model in mice fed the AHF and (2) to characterize resulting pain-related behaviors and pancreatic pathology. Histopathological confirmation of chronic alcoholic pancreatitis included the evidence of significant pancreatic fibrosis, fat vacuolization, and poor cellular architecture. Pain-related mechanical and heat hypersensitivity behavior were attenuated after antagonism with the peripherally restricted opiate, loperamide, in the mouse chronic pancreatitis model.

## 2. METHODS

The studies were performed in accordance with the Guide for the Care and Use of Laboratory Animals published by the National Institutes of Health. All procedures were approved by the University of Kentucky Institutional Animal Care and Use Committee.

### 2.1. Induction of Pancreatitis

Five months old male mice on a C57BL/6 background weighing less than 40 g (Jackson Laboratory, Bar Harbor, ME, USA) were used for this study. Animals were housed at 21–24°C, on a reverse light:dark 14:10 hour schedule. Control mice were fed rodent chow containing 10.4% fat (Mouse Breeder Diet 8626, Teklad, Madison, WI, USA). The mice with AHF pancreatitis were fed liquid diet (LD101A, Test Diet, Richmond, IN, USA) with added corn oil (3.3%, 33/1000 g) and supplementary lard given daily in a condiment dish (1 g). The total fat content was ~57%. Alcohol in the liquid diet was increased weekly from zero to 4%, 5%, and then 6%. The AHF fed mice were maintained at 6% alcohol for the remainder of the study. Body weight was monitored weekly.

### 2.2. Assessment of Pain Related Behaviors

#### 2.2.1. Paw Withdrawal Threshold Testing

Mice were placed on a raised Teflon wire-bottomed table in Plexiglas cubicles (7 × 4 × 4 cm) and acclimated for 30 min. Von Frey filaments ((4.74) 6.0g; (4.31) 2.0g; (4.08) 1.0g; (3.61) 0.4g; (3.22) 0.16g; (2.83)0.07g; (2.36) 0.02g; (1.65) 0.008g)) were applied to the plantar surface of the hind paws as previously described to determine the mechanical withdrawal threshold [17 – 20]. The decreased paw withdrawal threshold is an indication of mechanical hypersensitivity.

#### 2.2.2. Smooth or Rough Mechanical Plate

The test apparatus consisted of a clear Plexiglas box (11 × 8 × 8 cm) with either a smooth floor or a roughly textured floor insert. The rough insert was the textured side of a polystyrene ceiling light panel diffuser (Cat. # 89091, Lowes, Mooresville, NC, USA). Rearing exploratory activity, including frequency and duration, was monitored in real time and captured using custom software during 5 min tests.

#### 2.2.3. Modified 44°C Hotplate Assay

Mice were placed on a 38°C hotplate for 10 min to slowly pre-warm the animals’ feet. Animals were then placed on a 44°C hotplate for a 10 min testing period [21]. The number of jumps, rearing events, and latency to first jump were recorded using custom software.

### 2.3. Test Drugs

#### 2.3.1. Mu-opioid Receptor Agonist

Loperamide (Sigma-Aldrich, Milwaukee, WI, USA) was suspended in a 20% solution of 2-hydroxypropyl-β-cyclodextrin (Sigma-Aldrich) in saline and diluted to the proper dose concentration. Loperamide injections were made intraperitoneally (i.p.) at 0.4, 0.6, 0.8, or 1.2 mg/kg. Behavioral assays started 60 min post injection [1, 22].

### 2.4. Histology

Mice were perfused transcardially with 4% paraformaldehyde (PFA) and pancreata excised and post-fixed overnight, then changed to 70% ethanol. Pancreas tissues were embedded in paraffin, sectioned (10 μm) using a motorized microtome (Microm 350; Heidelberg, Germany), and mounted onto glass slides.

#### 2.4.1. Sirius Red Staining for Collagen

Pancreatic sections were deparaffinized, rehydrated, and stained with Sirius Red (0.1%; Electron Microscopy Sciences, Hatfield, PA, USA) and Fast Green counterstain. A Nikon Eclipse E1000 microscope was used with a 20x objective to capture random images of five sections per animal using ACT-1 software. Computer-assisted densitometry (NIH ImageJ) was used to calculate the percentage fibrotic tissue area stained red by Sirius Red.

#### 2.4.2. Hematoxylin and Eosin (H&E) Staining

Deparaffinized slides were rinsed in tap water and stained for 1 min with 0.1% hematoxylin (Fisher Scientific, Pittsburgh, PA, USA). Slides were washed, dehydrated, and stained for 1 min with 0.1% eosin before cover slipping with Permount (Fisher Scientific).

Photomicrographs of pancreas for each animal were taken from 5 randomly chosen sections and analyzed for morphological changes.

### 2.5. Statistical Analysis

The data are presented as means ± S.E.M. Comparisons among groups at different time points or different doses were performed with a two-way analysis of variance (ANOVA) followed by Tukey’s multiple comparisons post tests using SigmaPlot version 12.0 (Systat Software, San Jose California, USA). Two-tailed t-tests were used where appropriate. A *p*≤0.05 was considered significant.

## 3. RESULTS

### 3.1. Chronic AHF Fed Mice Had Increased Fibrosis and Histology Consistent With Chronic Pancreatitis

**Fig. (1).**
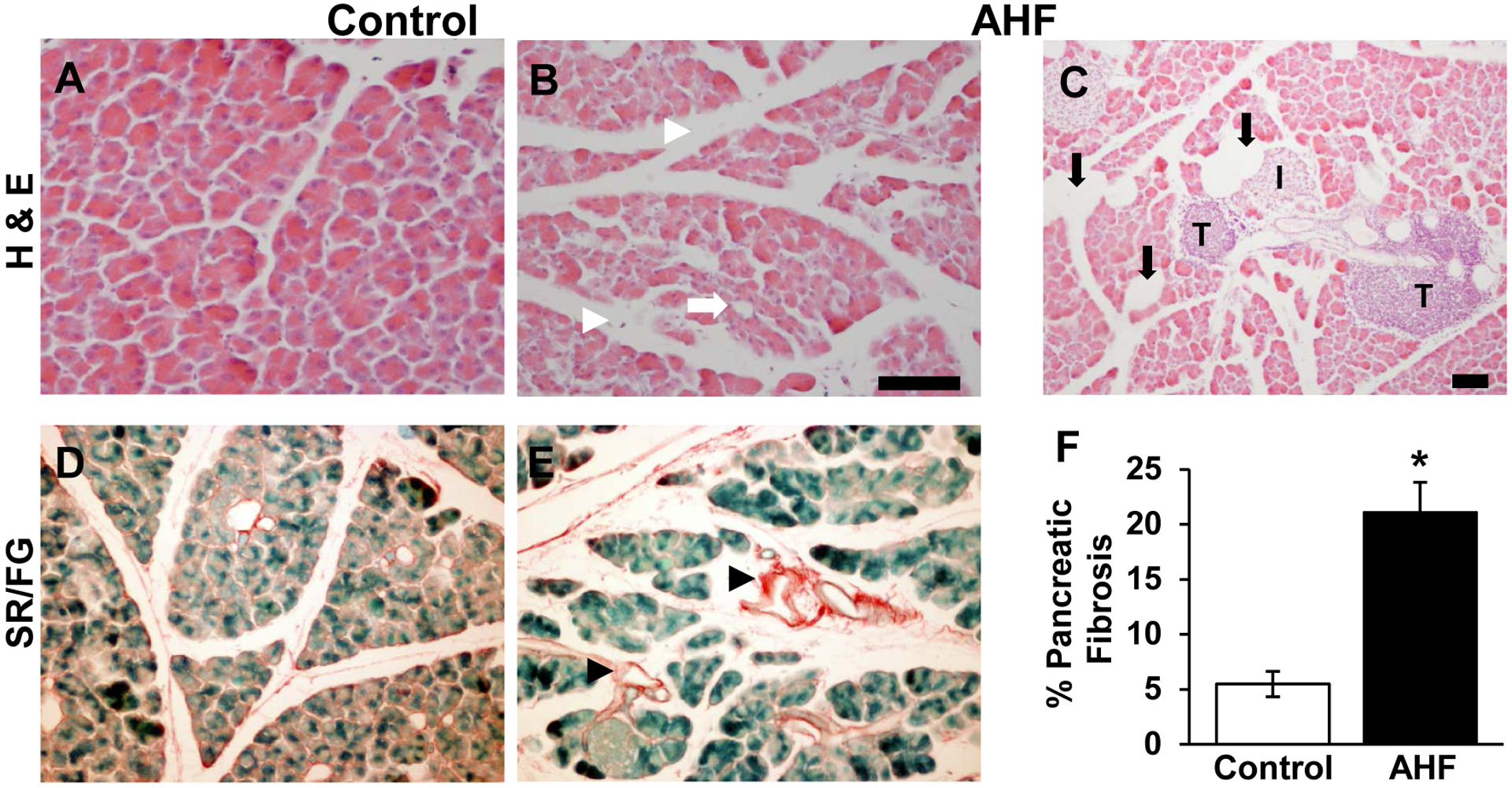
Histopathology Indicative of Chronic Pancreatitis Is Evident in Tissue Sections from AHF Fed Mice. (**A, D**) Pancreatic tissue sections from control mice fed standard rodent chow show normal structure morphology. (**B**) Increased intralobular spaces (white arrowheads), degradation of acinar cells (white arrow), (**C**) adipocytes (black arrows), tumor-like structures, and (**E**) fibrosis (black arrowheads) in pancreas sections from AHF fed mice. (**F**) Quantification of fibrosis determined there was a significant increase in percent of the total tissue area stained with Sirius Red in pancreas of AHF fed mice compared to controls (*p*<0.05, two-tailed t-test). (I: islets of Langerhans; T: tumor like structures; * *p*<0.05; Scale bars, 100 μm).

The pancreatic histology of AHF fed mice showed morphological disruption common to chronic pancreatitis: cellular atrophy, adipocytes, and increased intralobular spaces (Figs. 1B, C, E), compared to pancreatic tissues from control mice (Figs. 1A, D). Fatty infiltrations of lipid vacuoles were visible in pancreatic tissue of AHF fed mice. Both groups had tumor-like structures (Fig. 1C).

Significantly increased area of Sirius red stained fibrosis was identified in pancreas tissue samples from AHF fed mice compared to controls (Fig. 1F) (control: 5.5 ± 1.2%, AHF: 21.0 ± 2.7%; *p*<0.05, two-tailed t-test).

### 3.2. Animals Chronically Fed AHF Diet Developed Mechanical Hypersensitivity

**Fig. (2).**
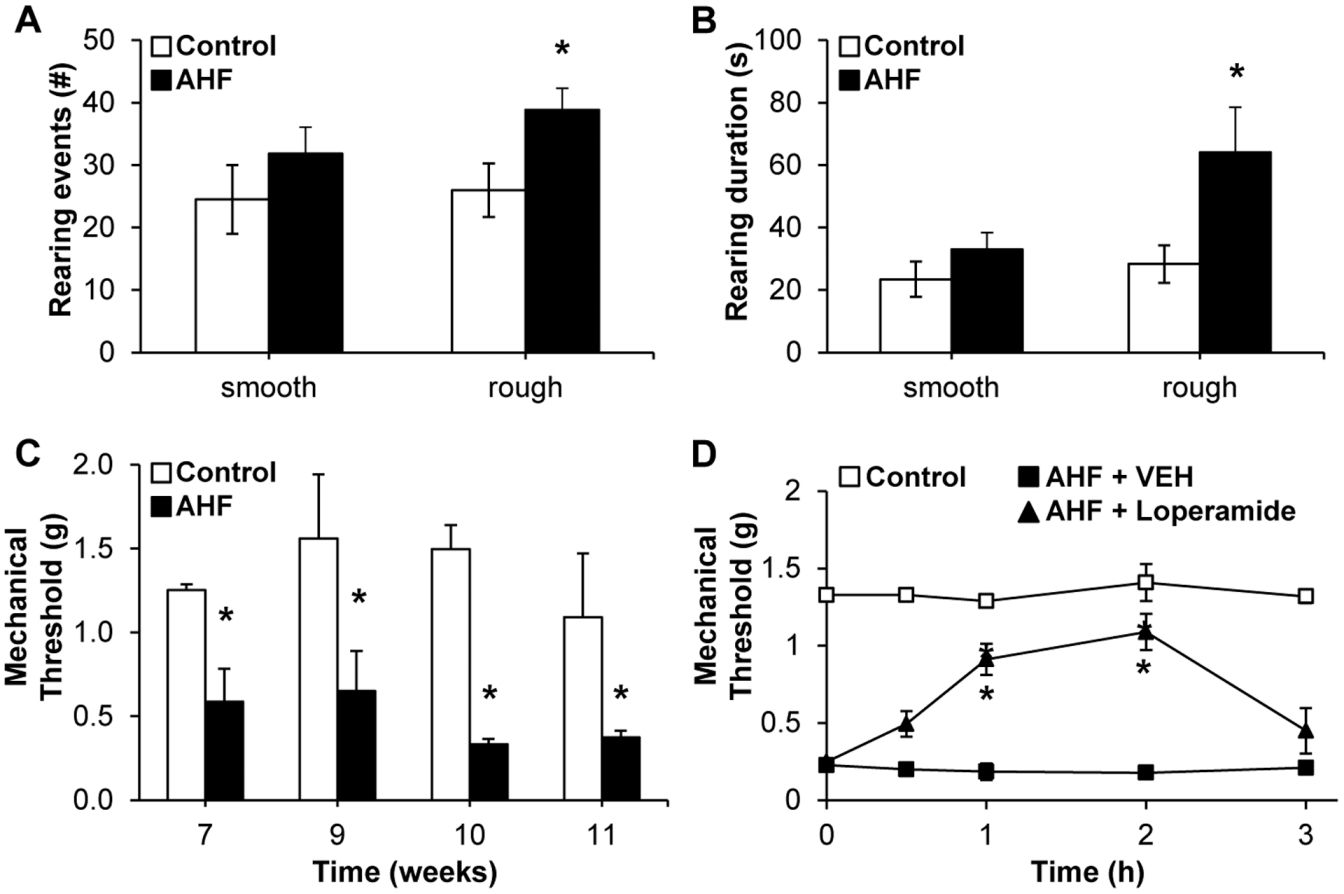
AHF Fed Animals Develop Mechanical Hypersensitivity. (**A**) Rearing events and (**B**) rearing duration were measured while animals were either on a smooth or rough surface. When animals were placed a rough surface, mice fed AHF diet reared significantly more often with increased rearing duration compared to animals fed control diet (*p*<0.05 by two-tailed t-test; Control n=7, AHF n=6). On a smooth surface, rearing events and duration were similar for both groups. (**C**) At chronic time points, week 7–12, paw withdrawal thresholds of AHF fed mice were significantly reduced compared to thresholds of control animals indicating hypersensitivity (p<0.05 by two-way ANOVA, Tukey post hoc test, n=6 per group). (**D**) Single low dose treatment with loperamide in week 12 significantly attenuated AHF-induced mechanical hypersensitivity within 1 h and mechanical sensitivity returned to pretreatment levels 3 hours post injection (*p*<0.05 by two-way ANOVA, Control n=4, AHF n=6). * *p*<0.05.

Secondary mechanical hypersensitivity was assessed by determining spontaneous escape behaviors (rearing events and rearing duration) on a smooth or rough surface as well as by testing hind paw withdrawal thresholds (Fig. 2). On a rough surface, the AHF diet fed animals displayed significantly more escape rearing behavior (AHF [n=6]: 38.8 ± 3.5; controls [n=7]: 26.0 ± 4.2; *p*<0.05 by two-tailed t-test). Likewise, rearing duration was significantly increased compared to controls (AHF: 64.1 ± 14.5 s; controls: 28.3 ± 6.0; *p*<0.05 by two-tailed t-test (Figs. 2A, B). No differences in spontaneous escape behavior were observed when animals were tested on the smooth surface (rearing events: AHF: 31.8 ± 4.2; controls: 24.5 ± 5.5; rearing duration: AHF: 33.0 ± 5.4s; controls: 23.5 ± 5.6 s). Similarly, hind paw mechanical withdrawal thresholds of AHF fed mice were significantly decreased (0.49 ± 0.16 g) compared to those of controls in von Frey fiber testing (1.33 ± 0.23 g; *p*<0.05 by two-way ANOVA, Tukey post hoc test) (Fig. 2C).

#### 3.2.1. Single Dose Loperamide Attenuated-Mechanical Hypersensitivity

Loperamide is a peripherally restricted mu-opioid agonist that is commonly used as an antidiarrheal agent. Animals fed AHF diet were given loperamide (0.6 mg/kg, i.p.) systemically and paw withdrawal thresholds determined (Fig. 2D). Mechanical hypersensitivity was significantly reversed at the 1 h (AHF + Loperamide [n=6]: 0.93 ± 0.33 g; AHF + VEH [n=4] 0.24 ± 0.10 g) and 2 h (AHF + Loperamide: 1.10 ± 0.35 g; AHF + VEH 0.15 ± 0.10 g) time points post injection, reverting to pre-injection levels by 3 hours.

### 3.3. AHF Fed Mice Developed Heat Hypersensitivity

**Fig. (3).**
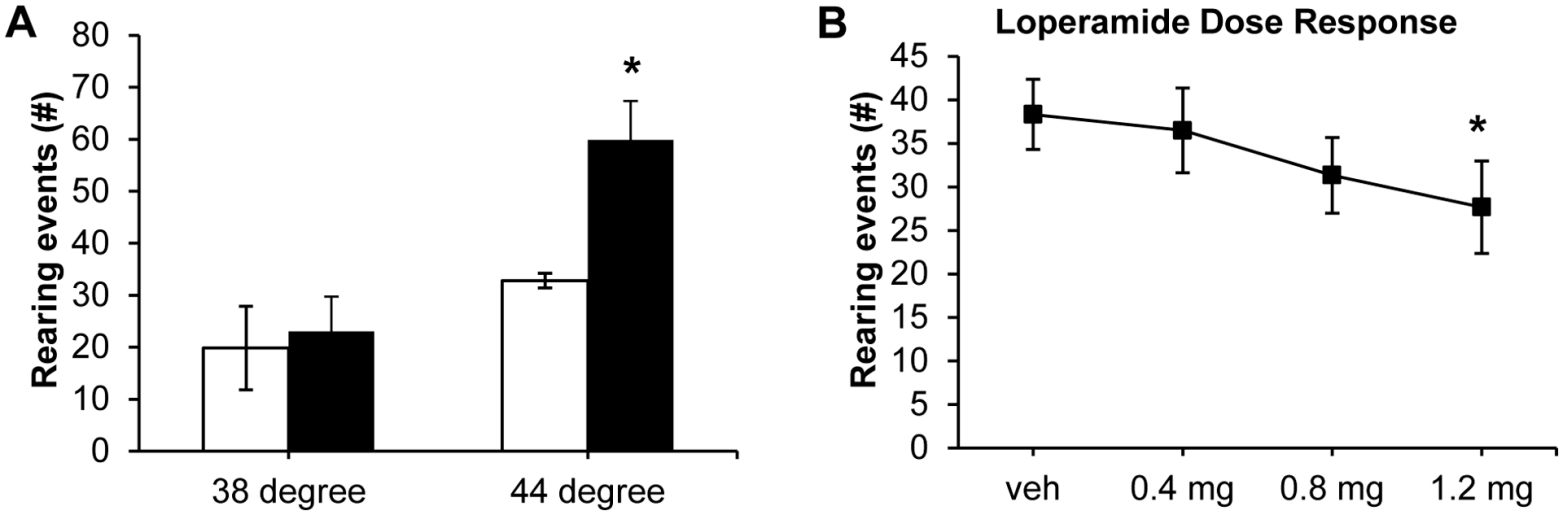
Heat Sensitivity of AHF Fed Mice on the Modified 44°C Hotplate Was Attenuated Dose Dependently by Loperamide. (**A**) At 44°C AHF fed mice reared significantly more often than control animals while at 38°C there was no difference. (**B**) Systemic treatment (i.p.) with loperamide dose dependently attenuated heat responses of AHF fed mice. The 1.2 mg/kg dose significantly reduced heat responses (*p*<0.05 by two-way repeated measures ANOVA). *p<0.05.

In the modified hotplate test animals were placed on the analgesiometer and the number of rearing events during a 5 min test period recorded to determine heat sensitivity. Using the modified 44°C hotplate test, AHF fed mice reared significantly more than control animals (AHF: 59.8 ± 7.5; control: 32.8 ± 1.4; *p*<0.05; by two tailed t-test) Fig. (3A). At 38°C no difference was detected between the two groups; mice fed AHF reared 23.0 ± 6.7 and control animals reared 19.8 ± 8.0 times.

#### 3.3.1. Loperamide Dose-Dependently Attenuated Nocifensive Responses in the 44°C Modified Hotplate Test

Mice fed AHF diet were treated with a single low dose of loperamide (i.p.) and heat sensitivity was measured 1 h later using the modified 44°C hotplate (Fig. 3B). Systemic loperamide decreased rearing events dose-dependently (0.4 mg/kg: 36.5 ± 4.9; 0.8 mg/kg: 31.3 ± 4.3 events; 1.2 mg/kg: 27.7 ± 5.3). Only the highest concentration of loperamide was able to significantly decrease the number of rearing events (*p*<0.05 by one-way ANOVA).

## 4. DISCUSSION

The present study demonstrated a non-invasive, alcohol and high fat diet induced mouse model of chronic pancreatitis that was produced in wildtype mice in the absence of noxious chemicals. The AHF diet model demonstrated histology and pain related behaviors closely resembling clinical symptoms of chronic pancreatitis as has been called for recently by the NCDD and the NIDDK [12]. In the clinic, up to 70% of patients with chronic pancreatitis are reported to abuse alcohol and the risk of pancreatitis is doubled in subjects who are overweight and obese [11, 23]. The AHF mouse model utilizes a diet that contains alcohol combined with high fat, thus, induces a “double hit” to the metabolism of the animal. This disrupts homeostasis, resulting in chronic inflammation of the pancreas and pain related behaviors.

Histological analysis of pancreas sections determined that only the AHF diet induced fibrosis, poor cellular architecture, and fat vacuole formation similar to our previous studies using rats [17–19,24]. The amount of fibrotic tissue in pancreas sections from AHF fed mice was significantly increased compared to age-matched wildtype samples. Increased fibrosis is a common feature in chronic pancreatitis caused by overactivation of pancreatic stellate cells (PSC), local immune cells in the pancreas [7]. In the presence of alcohol metabolites and excess fatty acids, normally quiescent PSCs become activated [24]. Persistent overactivation of PSCs causes excessive collagen production, which results in widespread pancreatic fibrosis and the characteristic damage seen in chronic pancreatitis. Increased fibrotic pancreas tissue in AHF fed mice is interpreted as an indicator of chronically overactivated PSCs in our mouse model.

Behavioral mechanical and heat hypersensitivity was determined in AHF fed mice after 7 weeks and lasted until experimental end. Animals fed AHF displayed significantly increased escape behavior from a rough testing surface and decreased paw withdrawal thresholds compared to controls. At the same time, AHF fed animals were more heat responsive, trying to escape the 44°C modified hotplate more often than control mice. Alcohol and fatty acid metabolic products activate not only PSCs but also directly sensitize nociceptors innervating the pancreas, thus producing pain related behaviors. In particular transient receptor potential (TRP) channels, which are expressed on PSCs as well as sensory neurons, have been identified as transducers of these metabolites [19, 24–27].

Almost all clinical patients with pancreatitis present with intractable abdominal pain and present pharmacological interventions have restricted efficacy and multiple side-effects including addiction. Here we investigated the efficacy of the peripherally restricted, non-addictive opioid loperamide [28, 29]. Loperamide is an opioid that does not cross the blood brain barrier and is one of the few opioids that is non-addictive [30]. It is best known for its use as an antidiarrheal medication, binding mu opioid receptors in the gastrointestinal system and causing constipation [31, 32]. In the present study, we found that systemic administration of loperamide attenuated both mechanical and heat hyperalgesia in our AHF mouse model of chronic pancreatitis at chronic time points. Similar to a previous study that used a kappa opioid receptor antagonist, analgesia was measured 1 h post injection [18]. The doses used were far below the recently described incidence of acute pancreatitis in a clinical patient after overdose [33]. The mu opioid receptor has been described in the pancreas, in particular in insulin producing β-cells, as well as in immune cells [34] and is widely expressed in pancreatic acinar cells after inflammation [35]. This opioid receptor is also expressed on primary sensory neurons and is upregulated after peripheral inflammation [36]. It is therefore not discernable if loperamide acted directly on nociceptors innervating the pancreas or indirectly by dampening the activity of pancreatic cells in this study. Studies have shown that loperamide inhibits pancreatic secretions both in rats [37] and in humans [38]. Future studies are needed to identify the exact mechanism.

Chronic pain is a significant problem for patients and one that is most important to address. Laboratory animal testing of analgesics has traditionally been limited to testing in acute pain models. The present study demonstrates a model with pain-related responses persisting at least through 10 weeks of study suitable for testing reflexive responses with stimuli such as the von Frey fiber and the hotplate tests. This model provides a stable backdrop for testing efficacy of therapeutics over many weeks. A second limitation of previous animal studies in acute models is that drugs that have been tested in laboratory animal trials do not always prove to be effective in treating clinical pain. The persisting pain-related responses in the AHF pancreatitis model are more comparable to the chronic clinical pain in patients with chronic pancreatitis. Identifying novel non-opiate analgesics for effective reduction of visceral pain signaling is a critical unmet clinical need for this syndrome with debilitating pain.

## CONCLUSION

The AHF diet is suitable to induce a chronic pancreatitis model in wildtype mice in the absence of noxious chemicals. Observed histopathology and behavioral hypersensitivity in our model are similar to clinical reports. This demonstrates that the AHF mouse model is suitable for the study of chronic pancreatic inflammation mechanisms and to identify novel pharmacological interventions at chronic time points.
